# Immune microenvironmental heterogeneity according to tumor DNA methylation phenotypes in microsatellite instability-high colorectal cancers

**DOI:** 10.1007/s00262-024-03805-3

**Published:** 2024-09-05

**Authors:** Jung Ho Kim, Jiyun Hong, Ji Ae Lee, Minsun Jung, Eunwoo Choi, Nam-Yun Cho, Gyeong Hoon Kang, Sangwoo Kim

**Affiliations:** 1 Department of Pathology, Seoul National University Hospital, Seoul National University College of Medicine, 101 Daehak-Ro, Jongno-Gu, Seoul, 03080 South Korea; 2 Cancer Research Institute, Seoul National University College of Medicine, Seoul, South Korea; 3 Department of Biomedical Systems Informatics, Yonsei University College of Medicine, Yonsei-Ro, Seodaemun-Gu, Seoul, 03722 South Korea; 4 Brain Korea 21 Project, Yonsei University College of Medicine, Seoul, South Korea; 5 Department of Pathology, Seoul National University Bundang Hospital, Seongnam, South Korea; 6 Department of Pathology, Severance Hospital, Yonsei University College of Medicine, Seoul, South Korea

**Keywords:** Colorectal carcinoma, CpG Island methylation, DNA methylation, Microsatellite instability, Mismatch repair, Tumor immunology

## Abstract

The detailed association between tumor DNA methylation, including CpG island methylation, and tumor immunity is poorly understood. CpG island methylator phenotype (CIMP) is observed typically in sporadic colorectal cancers (CRCs) with microsatellite instability-high (MSI-H). Here, we investigated the differential features of the tumor immune microenvironment according to CIMP status in MSI-H CRCs. CIMP-high (CIMP-H) or CIMP-low/negative (CIMP-L/0) status was determined using MethyLight assay in 133 MSI-H CRCs. All MSI-H CRCs were subjected to digital pathology-based quantification of CD3 + /CD8 + /CD4 + /FoxP3 + /CD68 + /CD204 + /CD177 + tumor-infiltrating immune cells using whole-slide immunohistochemistry. Programmed death-ligand 1 (PD-L1) immunohistochemistry was evaluated using the tumor proportion score (TPS) and combined positive score (CPS). Representative cases were analyzed using whole-exome and RNA-sequencing. In 133 MSI-H CRCs, significantly higher densities of CD8 + tumor-infiltrating lymphocytes (TILs) were observed in CIMP-H tumors compared with CIMP-L/0 tumors. PD-L1 TPS and CPS in CIMP-H tumors were higher than in CIMP-L/0 tumors. Next-generation sequencing revealed that, compared with CIMP-L/0 tumors, CIMP-H tumors had higher fractions of CD8 + T cells/cytotoxic lymphocytes, higher cytolytic activity scores, and activated immune-mediated cell killing pathways. In contrast to CIMP-L/0 tumors, most CIMP-H tumors were identified as consensus molecular subtype 1, an immunogenic transcriptomic subtype of CRC. However, there were no differences in tumor mutational burden (TMB) between CIMP-H and CIMP-L/0 tumors in MSI-H CRCs. In conclusion, CIMP-H is associated with abundant cytotoxic CD8 + TILs and PD-L1 overexpression independent of TMB in MSI-H CRCs, suggesting that CIMP-H tumors represent a typical immune-hot subtype and are optimal candidates for immunotherapy in MSI-H tumors.

**Supplementary Information:**

The online version contains supplementary material available at 10.1007/s00262-024-03805-3.

## Introduction

DNA methylation is an epigenetic mechanism that modulates gene expression without altering the DNA sequence [1]. DNA methylation can occur exclusively on cytosines of CpG dinucleotides. CpG islands, CpG-rich clusters, are commonly concentrated in the promoter region of genes and are generally maintained to be unmethylated in normal cells [1]. Promoter CpG island hypermethylation represses the expression of its corresponding gene, contributing to tumorigenesis by epigenetic silencing of tumor suppressor genes, similar to loss-of-function mutations in tumor suppressor genes [2]. The CpG island methylator phenotype (CIMP) is a distinct molecular subtype of tumors, which is characterized by extensive promoter CpG island methylation in multiple tumor-related genes. CIMP was first identified in a subset of colorectal cancers (CRCs) [3] and has been reported in various tumor types, although its frequency was generally low in non-CRC tumors [4].

CIMP is inversely correlated with genome-wide hypomethylation, such as long interspersed nucleotide element-1 (LINE-1) hypomethylation in CRCs [5]. Although the association between genome-wide methylation levels and immune evasion in tumors has been reported [6–8], there is a lack of studies on the association between CIMP and tumor immunity. Yates and Boeva analyzed the CIMP-associated features of various tumor types using The Cancer Genome Atlas datasets and suggested that CIMP might be associated with a specific tumor immune microenvironment (TIME) in various cancers, affecting immune cell composition and signatures [9]. This finding inspired us to study the link between CIMP and tumor immunity in CRCs.

CRCs can be largely classified into dichotomous CIMP subgroups based on the methylation frequencies of CIMP-specific gene promoter markers: CIMP-high (CIMP-H) and CIMP-low/negative (CIMP-L/0) subgroups [10, 11]. CIMP-H CRCs significantly overlap with CRCs with microsatellite instability-high (MSI-H) because most sporadic MSI-H CRCs develop through promoter CpG island methylation-induced silencing of *MLH1* gene, which is a frequent event in CIMP-H CRCs [12]. Therefore, among MSI-H CRCs, the majority of CIMP-H tumors can be regarded as sporadic cases, whereas most CIMP-L/0 tumors are caused by germline mutation in one of the DNA mismatch repair (MMR) genes, which is also referred to as Lynch syndrome [12]. Immunotherapy using immune checkpoint blockades (ICBs), such as programmed cell death protein 1 (PD-1) or programmed death-ligand 1 (PD-L1) inhibitors, has achieved great success in treating various types of cancers, and MSI-H has been established as a tissue-agnostic predictive biomarker for the therapeutic response to anti-PD-1/PD-L1 ICBs [13, 14]. Because of the high tumor mutational burden (TMB) observed in MSI-H tumors, they are characterized by heavy infiltration of cytotoxic T cells and upregulation of immune checkpoint molecules, which are strongly expected to respond to ICBs [13, 15]. However, not all patients with MSI-H CRC respond well to immunotherapy [14, 16]. One explanation for the limited response to ICBs in patients with MSI-H tumors is the intertumoral heterogeneity of TIME features [17]. According to our previous work, stratification of MSI-H CRCs into immune subgroups based on combined tumor-infiltrating lymphocyte (TIL) density and tertiary lymphoid structure (TLS) activity revealed significant differences in histologic features, activated signaling pathways, and gene expression subtypes between the immune-low and immune-high subgroups [18]. While our previous study revealed the heterogeneous immunologic features and the applicability of tailored therapeutic approaches based on genomic and transcriptomic immuno-molecular profiles [18], further stratification by CIMP status would warrant more complete investigation of the immunological and therapeutic heterogeneity in MSI-H CRCs.

Here, we comprehensively investigated the clinicopathologic characteristics, digital pathology-based TIME features, and next-generation sequencing (NGS)-based immuno-molecular profiles of a large series of human MSI-H CRC tissues, emphasizing differential TIME features depending on CIMP subgroups. We aimed to identify important clues regarding the direct or indirect interactions between CpG island methylation in tumor cells and tumor immune responses, excluding the potential confounding MSI-H effects.

## Materials and methods

### Tissue samples

Formalin-fixed, paraffin-embedded (FFPE) tissues from 133 MSI-H CRCs were retrospectively collected from the Pathology Archive of Seoul National University Hospital, Seoul, South Korea. All MSI-H CRC tissues were obtained from surgical specimens of patients who underwent surgical resection for CRC treatment at Seoul National University Hospital from 2014 to 2018. During this period, MSI testing was routinely performed for all surgically resected CRCs via fluorescence capillary electrophoresis-based DNA fragment analysis using five Bethesda microsatellite markers: BAT-25, BAT-26, D5S346, D17S250, and D2S123, as previously described [19]. A tumor that showed instability in two or more of the five microsatellite markers was considered MSI-H. Samples showing instability in dinucleotide repeat markers, but not in mononucleotide repeat markers, were excluded due to the possibility of false MSI-H [19]. Immunohistochemistry (IHC) for four DNA MMR proteins, including MLH1, MSH2, MSH6, and PMS2, was also performed for all MSI-H cases. Only samples showing both MSI-H and MMR deficiency (loss of expression of at least one MMR marker) were finally included in this study. Among the MSI-H CRCs, cases that underwent preoperative neoadjuvant chemotherapy and/or radiotherapy were excluded from the study cohort. This study complied with the ethical guidelines of the 2013 Declaration of Helsinki. All tissues included in this study were previously archived for research purposes in the Cancer Tissue Bank of Seoul National University Hospital, and informed consent was obtained from all patients. This study was approved by the Institutional Review Board of Seoul National University Hospital (IRB No. 1804–036-935).

### Clinicopathologic and molecular data collection

Clinical data were retrospectively collected from electronic medical records and survival registry data as previously described [17, 20]. Clinical information collected included age, sex, tumor location, gross tumor type, tumor size, American Joint Committee on Cancer/Union for International Cancer Control tumor-node-metastasis cancer stage, and disease-free survival (DFS) data. Histopathologic variables were microscopically evaluated by two independent gastrointestinal pathologists (JHK and JAL), and a consensus was reached. The histopathologic data included tumor grade (differentiation), lymphatic invasion, venous invasion, perineural invasion, mucinous histology, medullary histology, signet ring cell histology, tumor budding, poorly differentiated clusters, and desmoplastic reactions. Tumor grade was assessed as low or high based on the World Health Organization classification criteria of digestive tumors, 5th edition [21]. According to the International Tumor Budding Consensus Conference criteria, tumor budding was evaluated using the 3-tier scoring system [22]. Poorly differentiated clusters and desmoplastic reactions were assessed using Ueno’s criteria [23, 24]. *BRAF*/*KRAS* hotspot mutations were analyzed using Sanger sequencing as previously described [25]. Among the 133 MSI-H CRCs, two cases were excluded from *BRAF* mutation analysis because of the suboptimal quality or quantity of isolated tumor DNA samples.

### CIMP analysis

CIMP analysis was performed on 133 MSI-H CRC tissues, as previously described [26]. Briefly, genomic DNA was extracted from FFPE tumor tissues in each of the 133 MSI-H CRCs and subjected to bisulfite modification. Bisulfite-modified tumor DNA samples were analyzed by methylation-specific real-time PCR (MethyLight assay) using eight CIMP-specific gene promoter markers: *MLH1*, *CACNA1G*, *CDKN2A*, *CRABP1*, *IGF2*, *NEUROG1*, *RUNX3*, and *SOCS1*. The CIMP status in a tumor was dichotomously classified as CIMP-H (when five or more markers were methylated) or CIMP-L/0 (when four or fewer markers were methylated). A percentage of methylation reference > 4 indicated that the promoter CpG island locus was methylated.

### IHC

IHC for CD3, CD8, PD-L1, CD4, FoxP3, CD68, CD204, CD177, MLH1, MSH2, MSH6, and PMS2 in the 133 MSI-H CRC tissues was performed as previously described [17, 18, 20, 27]. IHC for CD3 (2GV6 clone; Ventana RTU, Roche, Basel, Switzerland), CD8 (SP57 clone; Ventana RTU, Roche), and PD-L1 (DAKO 22C3 clone; Agilent Technologies, Santa Clara, CA, USA) was performed using a representative whole-tumor slide for each case. IHC for CD4 (SP35 clone; Ventana RTU, Roche), FoxP3 (236A/E7 clone; Abcam, Cambridge, UK), CD68 (DAKO KP1 clone; Agilent Technologies), CD204 (SRA-E5 clone; Transgenic Inc., Fukuoka, Japan), CD177 (HPA041820 clone; Atlas antibodies, Bromma, Sweden), MLH1 (M1 clone; Ventana RTU, Roche), MSH2 (Invitrogen FE11 clone; ThermoFisher Scientific, Waltham, MA, USA), MSH6 (Cell Marque 44 clone; MilliporeSigma, Burlington, MA, USA), and PMS2 (Cell Marque MRQ-28 clone; MilliporeSigma) was conducted on multicore tissue microarray (TMA) sections of the 133 MSI-H CRCs. Multicore TMAs were constructed as described previously [18, 20]. Four TMA cores in each case were extracted from two different invasive margin (IM) and two different center of tumor (CT) areas. All IHC procedures, except PD-L1 IHC, were performed using the automated immunostainer Ventana BenchMark XT (Roche, Basel, Switzerland) or Bond-III (Leica Biosystems, Wetzlar, Germany). The PD-L1 22C3 IHC pharmDx assay was conducted on a representative whole-tumor slide of each case of the 133 MSI-H CRCs according to the manufacturer’s instructions using a DAKO Autostainer Link 48 (Agilent Technologies).

Detailed quantification methods of tumor-infiltrating immune cells (TIICs) and PD-L1 IHC are described in the next section (“Digital pathology-based TIME analysis”). MMR deficiency in a tumor was determined if at least one MMR IHC expression was completely negative in the nuclei of the tumor cells. Concurrent negativity for MLH1/PMS2 or MSH2/MSH6 indicated MLH1 or MSH2 deficiency, respectively. Isolated MSH6 or PMS2 expression loss indicated MSH6 or PMS2 deficiency, respectively. Lymphocyte nuclei in and around the tumor cells were used as positive controls for MMR IHC expression.

### Digital pathology-based TIME analysis

Quantitative analysis of various TIME features using digital pathology analysis of IHC or hematoxylin and eosin-stained (H&E) slides of the 133 MSI-H CRCs was performed as previously described [17, 18, 20]. Detailed methods for quantification of densities of TIICs in the IM and CT areas and histomorphometric analysis of TLS are thoroughly described in our previous reports [18, 20]. PD-L1 IHC expression was quantitatively evaluated by two experienced pathologists (JHK and JAL) using two widely used scoring systems: tumor proportion score (TPS) and combined positive score (CPS), as previously described [28]. Briefly, the PD-L1 TPS was calculated as the number of PD-L1-positive tumor cells divided by the total number of PD-L1-positive and -negative tumor cells on a PD-L1-stained whole slide in each case. The PD-L1 CPS was also assessed on a PD-L1-stained whole slide in each case and was calculated from the number of PD-L1-positive cells, including tumor and immune cells, divided by the total number of viable tumor cells, and multiplied by 100.

### Whole-exome and RNA-sequencing

A stepwise selection of samples for NGS analysis is summarized in Supplementary Fig. S1. NGS analysis was performed on 33 representative MSI-H CRCs selected based on strict criteria for quality and quantity of paired tumor-normal fresh frozen tissues and practical considerations. Twelve CIMP-H (36%) and 21 CIMP-L/0 (64%) cases for NGS analysis were finally selected to maintain their proportional distributions similar to those in our MSI-H CRC cohort (45 CIMP-H (34%) and 88 CIMP-L/0 (66%) cases among the 133 MSI-H CRCs) (Supplementary Fig. S1). For whole-exome sequencing, 0.1‒0.5 μg of fragmented DNA was used with the SureSelect Human All Exon Kit V5. This process involved random fragmentation, adapter ligation, purification, hybridization, and PCR amplification. The quality of the captured libraries was assessed using an Agilent 2100 Bioanalyzer (Agilent Technologies). Sequencing was performed using the NovaSeq 6000 system. For RNA-sequencing, 100 ng of total RNA was used for library preparation following the TruSeq stranded total RNA kit protocol. This included ribosomal RNA depletion and strand-specific methodologies. Library quality was evaluated using an Agilent 2100 Bioanalyzer, and quantification was performed using the KAPA library quantification kit (Kapa Biosystems, MA, USA). Sequencing was performed using a NovaSeq 6000 system.

### Genomic data analysis

Quality assessment, and preprocessing of low-quality read were performed on all reads using fastp v0.21.0. The remaining reads were aligned to the human reference genome (GRCh38) using BWA-MEM v0.7.17 (for DNA) [29] and STAR v.2.7.3a in two-pass mode (for RNA) [30]. For genomic variant calling, base quality was corrected using the BaseRecalibrator and ApplyBQSR modules of GATK [31]. Somatic variants were selected, and artifacts were filtered out using Mutect2 with matched normal DNA and public databases [31]. The remaining genomic variations were annotated to elucidate their effects using the Ensembl Variant Effect Predictor [32], followed by conversion into MAF format using vcf2maf v1.6.20. Subsequently, our downstream genomic analysis focused exclusively on non-synonymous genomic variants. TMB was quantified based on nonsynonymous somatic mutations per megabase (Mb).

### Transcriptomic data analysis

For transcriptomic data analysis, read counts were used for gene quantification and measured using HTSeq v0.11.1 [33]. Low-expressed genes were excluded to mitigate bias, and batch effects were adjusted using Combat-seq [34]. We then obtained a normalized gene expression matrix and identified differentially expressed genes (DEGs) using the DESeq2 R package v1.26.0 [35]. The selection of DEGs adhered to the following criteria: (1) adjusted *p*-value < 0.05 and (2) absolute Log2 fold change > 2. Additionally, the normalized expression matrix facilitated the determination of consensus molecular subtypes (CMS) of colorectal cancer. The classification was performed using the nearest-centroid single sample predictor algorithm in the CMSclassifier R package v1.0.0 [36].

### Immunogenic activity profiling

Immune cell deconvolution was implemented using the immunoedeconv R package v2.1.0 [37]. The cytolytic activity (CYT) score was gauged by calculating the geometric mean expression of Granzyme A (*GZMA*) and Perforin 1 (*PRF1*), as indicated in the original study [38].

### Gene set enrichment analysis

Gene set enrichment analysis (GSEA) was performed using the GSVA R package v1.44.5 [39] and clusterProfiler R package v3.14.3 [40]. Reference databases were obtained using Msigdbr v7.5.1.

### Statistical analysis

All statistical analyses in this study were performed using the SPSS version 23 (IBM, Armonk, NY, USA) and GraphPad Prism version 10 (GraphPad Software, San Diego, CA, USA). Parametric or non-parametric categorical variables were compared using the chi-square or Fisher’s exact test, respectively. A mean comparison between parametric or non-parametric continuous variables was conducted using the Student’s t-test or Mann–Whitney U test, respectively. Survival analysis of DFS data was performed using the Kaplan–Meier method with a log-rank test. In the NGS analyses, the statistical significance of *p*-values for the normalized enrichment score was assessed using a permutation test. All *p*-values were two-sided, and statistical significance was determined if a *p*-value was less than 0.05. The *p*-values of cell-type specific mean expression from six algorithms used during cell deconvolution were combined by Fisher method.

## Results

### Clinicopathologic characteristics according to CIMP status in MSI-H CRCs

The clinical, pathologic, and molecular features of the 133 MSI-H CRCs are summarized in Supplementary Table S1. Compared with CIMP-L/0 tumors, CIMP-H tumors were significantly associated with older age (≥ 64 years; 87% vs. 44%; *p* < 0.001), female sex (76% vs. 39%; *p* < 0.001), right-sided tumor location (91% vs. 70%; *p* = 0.007), medullary histology (29% vs. 9%; *p* = 0.003), MLH1 expression loss (100% vs. 47%; *p* < 0.001), *KRAS* mutation absence (93% vs. 50%; *p* < 0.001), and *BRAF* V600E mutation presence (27% vs. 0%; *p* < 0.001). These features are consistent with the known characteristics of CIMP-H CRCs [41] and indicate that there may be a minimum demographic bias in our sample cohort. In the Kaplan–Meier survival analysis, no difference in DFS between the CIMP-H and CIMP-L/0 subgroups was observed in the 133 patients with MSI-H CRC (log-rank *p* = 0.897; Supplementary Fig. S2).

### Digital pathology-based TIME characteristics according to CIMP status in MSI-H CRCs

**Fig. 1:**
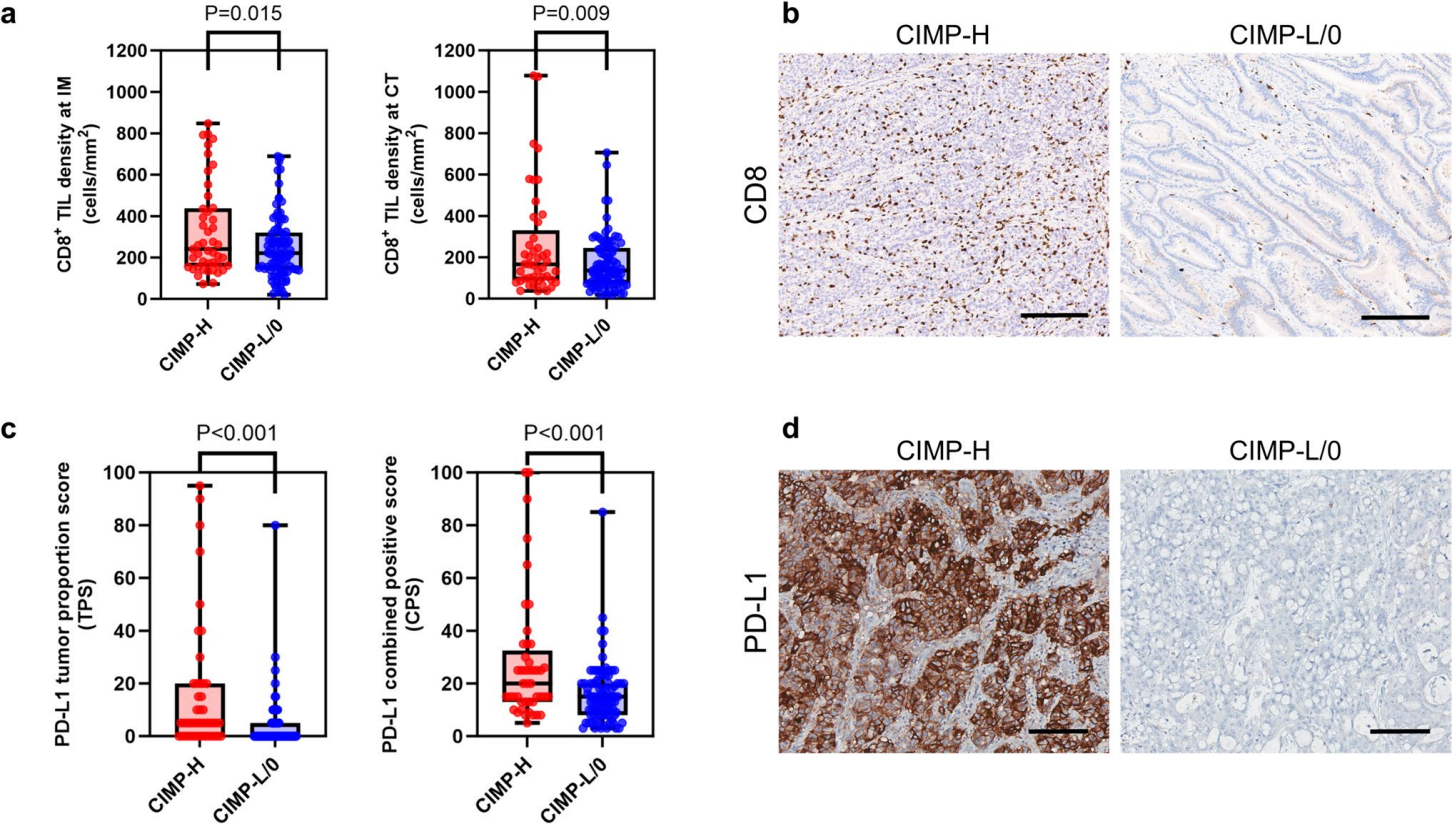
Comparison of pathology-based TIME features between CIMP subgroups of MSI-H CRCs. **a** Comparison of CD8^+^ TIL densities between CIMP-H and CIMP-L/0 subgroups of MSI-H CRCs at IM (left) and CT (right) areas. **b** Representative photomicrographs of CD8 IHC in CIMP-H and CIMP-L/0 MSI-H CRCs. Note the higher density of CD8^+^ TILs in the CIMP-H tumor, compared with the CIMP-L/0 tumor (scale bar, 200 μm). **c** Comparison of PD-L1 IHC scores, including TPS (left) and CPS (right), between CIMP-H and CIMP-L/0 subgroups of MSI-H CRCs. **d** Representative photomicrographs of PD-L1 IHC in CIMP-H and CIMP-L/0 MSI-H CRCs. Note the diffuse strong expression pattern of PD-L1 in the CIMP-H tumor, compared with the CIMP-L/0 tumor (scale bar, 100 μm). Abbreviations: TIME, tumor immune microenvironment; CIMP, CpG island methylator phenotype; CIMP-H, CIMP-high; CIMP-L/0, CIMP-low/negative; MSI-H, microsatellite instability-high; CRCs, colorectal cancers; TIL, tumor-infiltrating lymphocyte; IM, invasive margin; CT, center of tumor

We comprehensively evaluated the TIME features of 133 MSI-H CRCs using digital pathology-based quantification of the densities of TIICs in the IM and CT areas, TLS diameters, and PD-L1 expression on IHC- or H&E-stained whole slides. The TIICs assessed included CD3 + TILs, CD8 + TILs, CD4 + TILs, FoxP3 + TILs, CD68 + TAMs, CD204 + TAMs, and CD177 + TANs. Among the TIME parameters, only CD8 + TILs and PD-L1 expression were significantly associated with the CIMP status in MSI-H CRCs (Fig. 1). Compared with CIMP-L/0 tumors, CIMP-H tumors correlated with higher densities of CD8 + TILs in the IM and CT areas (*p* = 0.015 and *p* = 0.009, respectively; Fig. 1a, b). However, the other TIICs, including CD3 + TILs, CD4 + TILs, FoxP3 + TILs, CD68 + TAMs, CD204 + TAMs, and CD177 + TANs, did not differ according to CIMP status in MSI-H CRCs (Supplementary Fig. S3a-d and Supplementary Fig. S4a-c). Peritumoral TLS activity in each case was assessed using Ueno’s criteria (maximum diameter of the largest TLS), and there were no significant differences in the maximum diameters of TLSs or frequencies of active TLS subgroup (maximum diameter of TLS > 1 mm) according to the CIMP status in MSI-H CRCs (Supplementary Fig. S3d). PD-L1 IHC expression was evaluated using the TPS and CPS criteria, and PD-L1 TPS and CPS were significantly different between the CIMP-H and CIMP-L/0 subgroups of MSI-H CRCs (both *p* < 0.001; Fig. 1c, d).

### NGS-based immuno-molecular characteristics according to CIMP status in MSI-H CRCs

**Fig. 2:**
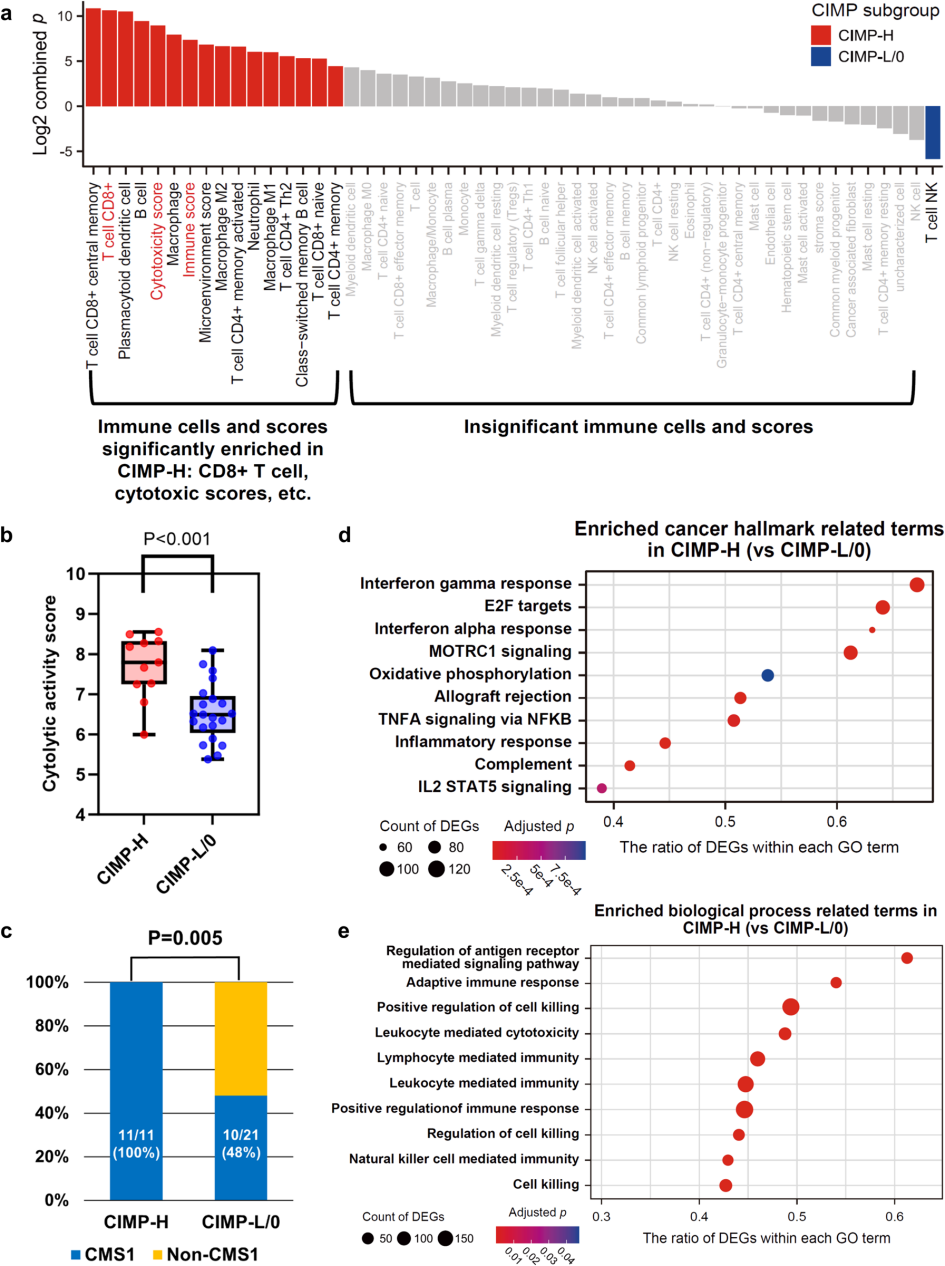
Comparison of transcriptome-based immuno-molecular features between CIMP subgroups of MSI-H CRCs. **a** Comparison of immune cell types and immune-related scores between CIMP-H and CIMP-L/0 subgroups of MSI-H CRCs. The combined *p*-value is computed using the Fisher method. Colors indicate significance and enrichment: Red signifies significantly enriched cell types and scores in CIMP-H tumors, while blue signifies significantly enriched cell types and scores in CIMP-L/0 tumors. Gray indicates insignificant cell types and scores. **b** Comparison of cytolytic activity scores between CIMP-H and CIMP-L/0 subgroups of MSI-H CRCs. **c** Comparison of frequencies of CMS1 between CIMP-H and CIMP-L/0 subgroups of MSI-H CRCs. **d** GSEA-based comparison of 50 cancer hallmarks between CIMP-H and CIMP-L/0 subgroups of MSI-H CRCs. The dot size corresponds to the gene counts associated with gene ontology terms, while the color gradient from purple to red indicates the adjusted *p*-value. **e** GSEA-based comparison of biological process terms between CIMP-H and CIMP-L/0 subgroups of MSI-H CRCs. Abbreviations: CIMP, CpG island methylator phenotype; CIMP-H, CIMP-high; CIMP-L/0, CIMP-low/negative; MSI-H, microsatellite instability-high; CRCs, colorectal cancers; CMS1, consensus molecular subtype 1; GSEA, gene set enrichment analysis; DEG, differentially expressed gene; GO, gene ontology

**Fig. 3:**
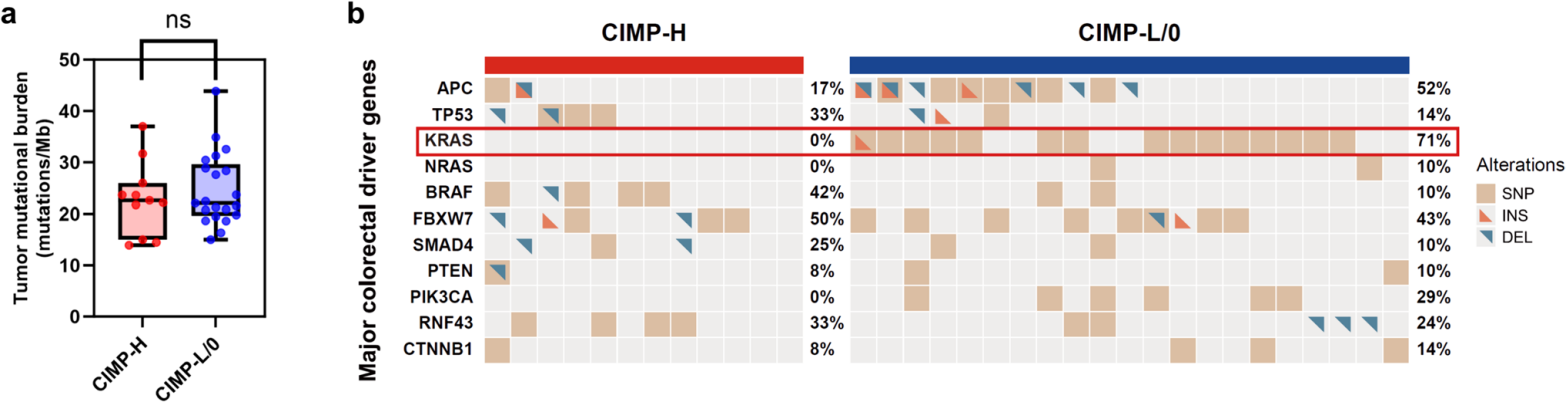
Comparison of genomic features between CIMP subgroups of MSI-H CRCs. **a** Comparison of TMB between CIMP-H and CIMP-L/0 subgroups of MSI-H CRCs. **b** The mutational landscape comparison between CIMP-H and CIMP-L/0 subgroups of MSI-H CRCs. Genes include known major oncogenes or tumor suppressor genes in CRC. Alteration types were denoted by color and shape. Abbreviations: CIMP, CpG island methylator phenotype; CIMP-H, CIMP-high; CIMP-L/0, CIMP-low/negative; MSI-H, microsatellite instability-high; CRCs, colorectal cancers; TMB, tumor mutational burden; ns, not significant

To understand the genomic and transcriptomic basis of TIME heterogeneity according to CIMP status in MSI-H CRCs, we conducted whole-exome sequencing and bulk RNA-sequencing analyses in 33 representative cases of MSI-H CRCs (12 CIMP-H and 21 CIMP-L/0). Transcriptome-based immuno-molecular features and genomic features according to CIMP status in MSI-H CRCs are summarized in Figs. 2 and 3, respectively. Unsupervised clustering of immune cell deconvolution analysis using RNA-sequencing revealed that CIMP subgroups are distinguished by comprehensive TIME features (Cluster 1: 19/25 CIMP-L/0 vs. Cluster 2: 6/8 CIMP-H; *p* = 0.015; Supplementary Fig. S5). We observed higher abundances of CD8 + T cells (combined *p* = 0.0006), cytotoxicity lymphocytes (*p* = 0.002), and immune score (*p* = 0.006) in CIMP-H tumors compared to CIMP-L/0 tumors (Fig. 2a). Additionally, significantly higher CYT scores were observed in CIMP-H tumors than in CIMP-L/0 tumors (*p* < 0.001; Fig. 2b). In the transcriptome-based CRC molecular subtyping analysis, CMS1, the typical MSI-immune subtype, was significantly enriched in CIMP-H tumors (100%) compared with CIMP-L/0 tumors (48%) (*p* = 0.005; Fig. 2c). Immune pathways of cancer hallmarks, including interferon alpha and gamma responses, allograft rejection, complement, and inflammatory response, were significantly enriched in CIMP-H tumors compared with CIMP-L/0 tumors (Fig. 2d). Furthermore, a set of immune-related biological pathways were co-enriched in CIMP-H tumors, indicating higher activity of cytotoxic/cytolytic immunity (Fig. 2e), without differences in TMB (number of non-synonymous mutations/MB = 9.62‒37.60 vs. 16.06‒47.84 in CIMP-H vs. CIMP-L/0; *p* = 0.326) (Fig. 3a). Among major CRC driver genes, *KRAS* mutation was enriched and exclusive found in CIMP-L/0 (n = 15/21; 71%), compared to CIMP-H (n = 0/12; 0%) (adjusted *p* < 0.001) (Fig. 3b).

## Discussion

Although the importance of TIME in cancer prognosis and treatment has been recognized and vigorously studied, there has been a lack of consistent results regarding the impact of tumor DNA methylation on tumor immunity. Jung et al. showed that the global methylation loss correlated with immune evasion signatures in cancers [6]. Park et al. demonstrated that methylation burden was negatively correlated with CYT score in various cancer types [7]. Johnstone et al. reported that DNA hypomethylation was linked to spatial compartmental reorganization of tumor genome and could have tumor-suppressive effects by downregulating genes associated with epithelial-mesenchymal transition, invasion, and stemness and upregulating genes with pro-immunity functions [8]. Although these studies mainly investigated the methylation levels of genome-wide CpG loci, mostly encompassing methylation in repetitive sequences such as LINE-1, the exact association between CIMP and tumor immunity has been rarely studied. Recently, Yates and Boeva analyzed the immune composition of multiple tumors that exhibited CIMP and found that specific immune cell types were enriched in the CIMP-H subtype of tumors, especially in CRC, tumor-infiltrating cytotoxic CD8 + T cells were correlated with CIMP-H status [9]. However, this observation may be based on an indirect effect by MSI-H, as CIMP-H and MSI-H significantly overlap in CRCs, commonly mediated by *MLH1* promoter methylation [12]. MSI-H tumors generally display an immunogenic status, including increased TILs and active TLSs, owing to their high neoantigen loads resulting from high TMB [15]. Therefore, to uncover the direct impact of CIMP on tumor immunity in CRC, it was necessary to control for the MSI-H factor in the study samples. Thus, we decided to collect a large series of MSI-H CRCs to exclude the confounding effects of MSI-H. In this study, by subgrouping MSI-H CRCs into CIMP-H and CIMP-L/0, we investigated the association between CIMP and TIME in CRC.

**Fig. 4:**
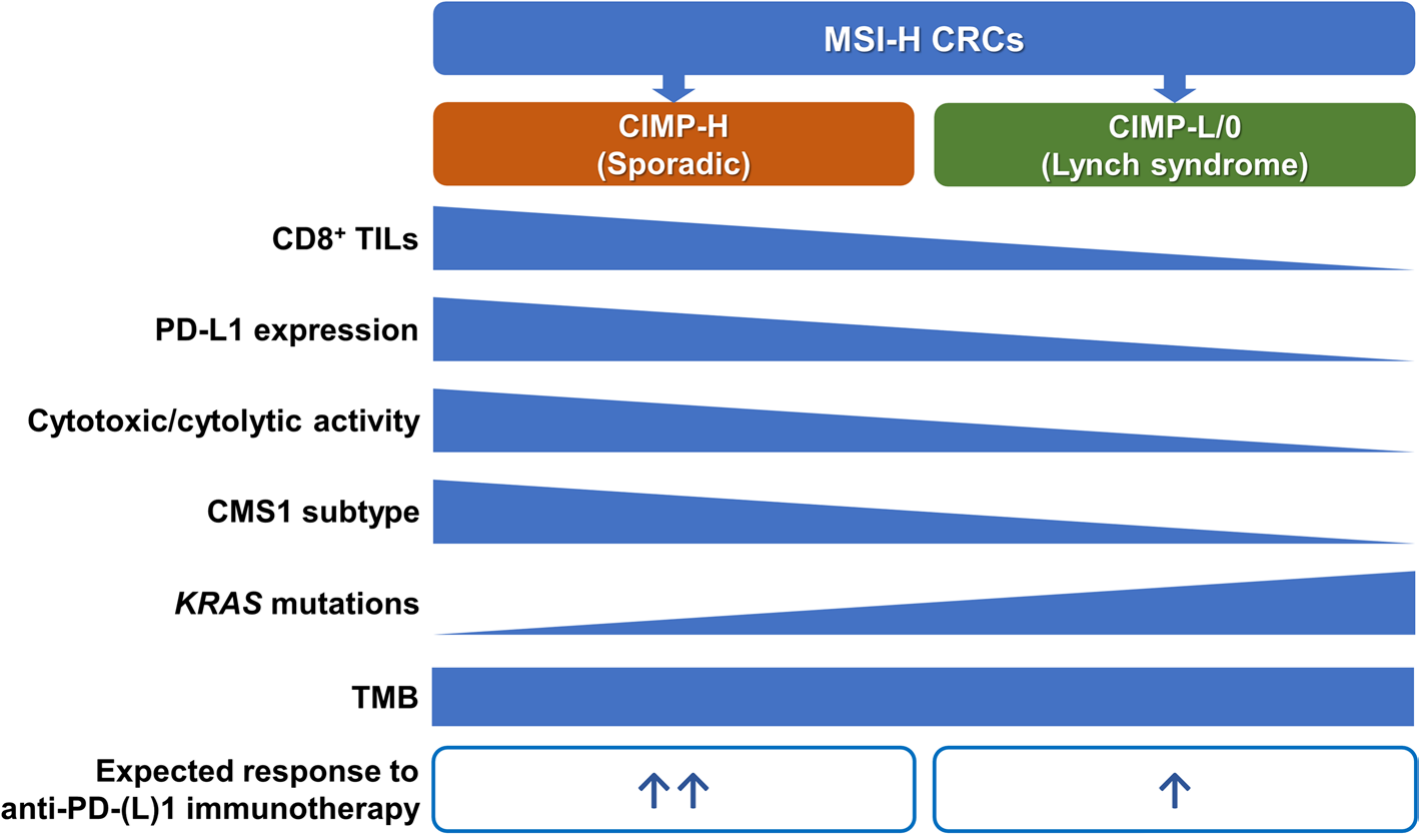
Graphical summary of this study

We found that cytotoxic CD8 + TILs and cytolytic activity scores were higher in the CIMP-H subgroup compared with the CIMP-L/0 subgroup in the MSI-H CRCs. Consistent with these features, CMS1, a typical immune-high transcriptomic subtype of CRC, was significantly enriched in the CIMP-H subgroup of MSI-H CRCs (Fig. 2c). These findings imply that tumor-suppressive anti-tumor immunity is stronger in CIMP-H tumors than in CIMP-L tumors. As suggested in our previous study [27], PD-L1 protein expression scores in tumor and immune cells were significantly higher in CIMP-H tumors than in CIMP-L tumors in MSI-H CRCs (Fig. 1c). The combination of high density of cytotoxic CD8 + TILs and PD-L1 overexpression indicates a potential response to anti-PD-1/PD-L1 immunotherapy in various cancers [42]. Although all MSI-H CRCs are generally regarded as good candidates for immunotherapy, our data shows that the CIMP-H subgroup of MSI-H CRCs can be considered more optimal for anti-PD-1/PD-L1 immunotherapy than the CIMP-L subgroup (Fig. 4).

According to our data, although differences in tumor immunity were found between CIMP-H and CIMP-L/0 subgroups in MSI-H CRCs, there was no difference in DFS according to CIMP status in patients with MSI-H CRC. However, this does not indicate a lack of clinical significance in our findings. The DFS data used in our retrospective MSI-H CRC cohort reflected tumor recurrence or patient’s death after radical surgery and did not indicate outcomes after immunotherapy. Differences in TIME features according to CIMP status in MSI-H CRCs are not necessarily linked to the patient’s natural prognosis, but our study provides important clues that the CIMP-H subgroup may be more responsive to immunotherapy than the CIMP-L/0 subgroup among patients with MSI-H CRC. Therefore, further clinical studies will be needed to determine whether there is a real difference in the immunotherapy response according to CIMP status in cancers. In addition, even if the findings of this study do not immediately change clinical practice, they can be expected to be indirectly used in various ways in the advancement of the precise selection of immunotherapy candidates among cancer patients, the development of novel immunotherapy targets related to tumor DNA methylation characteristics, and the development of effective combination treatment strategies such as a combination of immunotherapy and epigenetic drugs.

We wondered whether differential TIME features according to CIMP status in MSI-H CRCs might be based on differential tumor genetic factors that affect tumor immunity. High TMB is closely associated with augmented anti-tumor immunity due to high tumor-specific neoantigen loads in various cancers [15], and mutations in immune cycle-related genes such as genetic mutations disrupting the antigen presentation machinery in tumor cells can induce immune evasion in tumors [43]. In addition, *KRAS* mutations have been suggested as one of the immuno-suppressive genetic factors in CRCs [44]. Thus, we explored the underlying genetic factors potentially affecting tumor immune responses in our MSI-H CRC samples using NGS analysis. As a results, TMB levels were not significantly different between CIMP-H and CIMP-L/0 tumors in MSI-H CRCs (Fig. 3a). This finding suggests that hypermutated characteristics in MSI-H CRCs are preserved regardless of CIMP status and that differences in TIME features according to CIMP status in MSI-H CRCs may not be based on differences in TMB. Then, when comparing the mutation frequencies of major cancer driver and immune evasion-related genes between the CIMP subgroups in MSI-H CRCs, *KRAS* mutation was the only significant factor (Fig. 3b and Supplementary Table S1). To elucidate whether *KRAS* mutations were responsible for the differential TIME features according to the CIMP status in MSI-H CRCs, we compared CD8 + TIL densities and PD-L1 expression scores according to *KRAS* mutation status in MSI-H CRCs. There were no significant differences in CD8 + TIL densities and PD-L1 expression scores between the CIMP subgroups, except for the PD-L1 CPS score, which showed marginal significance (Supplementary Fig. S6). These results suggest that the increased cytotoxic anti-tumor immunity and PD-L1 expression observed in CIMP-H tumors may not be due to a lack of *KRAS* mutations. These findings indicate that the significant associations between CIMP and tumor immunity in MSI-H CRCs may not be an indirect consequence of the direct effect of other underlying genetic factors. Recent evidence from Tricarico et al. supports that CIMP-H is directly associated with higher tumor immunity in experimental models of CRC [45]. The authors suggested that the methylation/demethylation imbalance in CIMP-H tumors may fundamentally contribute to the pro-inflammatory characteristics, which are then intensified by the MSI-H status during progression to CRCs with typically overlapping CIMP-H/MSI-H [45]. Further analyses are necessary to determine whether there are similar associations between CIMP and TIME in non-MSI-H (microsatellite stable) CRCs and various tumor types other than CRCs.

Our study had several limitations. First, we only conducted observational analyses using human tumor tissues and did not perform functional experiments to uncover the mechanism by which CIMP-H tumors are more heavily infiltrated by cytotoxic CD8 + TILs and have more active cytolytic immunity than CIMP-L/0 tumors. Nevertheless, we excluded the potential confounding effects of TMB or major genetic mutations on differential TIME features according to CIMP status in MSI-H CRCs, suggesting that CIMP-H may be directly associated with increased immune responses in tumors. More basic and translational studies are needed to better understand the mechanisms underlying immune modulation by CpG island methylation in tumor cells. Second, we could not demonstrate the clinical therapeutic evidence relevant to our findings. Although CIMP-H tumors are conceptually expected to be more responsive to anti-PD-1/PD-L1 immunotherapy than CIMP-L/0 tumors, our MSI-H CRC cohort was retrospectively collected, and only a few patients received immunotherapy using ICB. Future clinical studies are required to address the immunotherapeutic relevance of the CIMP status in patients with MSI-H CRC.

In conclusion, the CIMP-H subgroup of MSI-H CRCs is characterized by increased infiltration of cytotoxic TILs and PD-L1 overexpression and thus can be expected to respond well to immunotherapy. CIMP-associated TIME features in MSI-H CRCs may be independent of the immunologic effect of TMB or specific mutations. Potential direct interactions between tumor DNA methylation and tumor immunity should be further explored in various tumors.

## Supplementary Information

Below is the link to the electronic supplementary material.

Supplementary file1 (PDF 1154 KB)

Supplementary file2 (PDF 177 KB)

## Data Availability

The raw dataset generated from whole-exome sequencing and RNA-sequencing during the current study are available in the Sequence Read Archive (SRA) under the accession number PRJNA1035153 (https://www.ncbi.nlm.nih.gov/sra/?term=PRJNA1035153). The other data generated in the study are available from the corresponding authors upon reasonable request.

## Abbreviations

CIMP: CpG island methylator phenotype
CMS: Consensus molecular subtype
CPS: Combined positive score
CRC: Colorectal cancer
CT: Center of tumor
CYT: Cytolytic activity
DEG: Differentially expressed gene
DFS: Disease-free survival
FFPE: Formalin-fixed, paraffin-embedded
GSEA: Gene set enrichment analysis
ICB: Immune checkpoint blockade
IHC: Immunohistochemistry
IM: Invasive margin
MMR: Mismatch repair
MSI: Microsatellite instability
NGS: Next-generation sequencing
PD-1: Programmed cell death protein 1
PD-L1: Programmed death-ligand 1
TIIC: Tumor-infiltrating immune cell
TIL: Tumor-infiltrating lymphocyte
TIME: Tumor immune microenvironment
TMA: Tissue microarray
TMB: Tumor mutational burden
TLS: Tertiary lymphoid structure
TPS: Tumor proportion score
