# Supplementary material for: Immune microenvironmental heterogeneity according to tumor DNA methylation phenotypes in microsatellite instability-high colorectal cancers
Source: Cancer Immunol Immunother. 2024 Sep 5;73(11):215. doi: 10.1007/s00262-024-03805-3 (PMC11377388; doi:10.1007/s00262-024-03805-3)
Supplement: Supplementary file 1 — Supplementary file1 (PDF 1154 KB) [file 262_2024_3805_MOESM1_ESM.pdf]

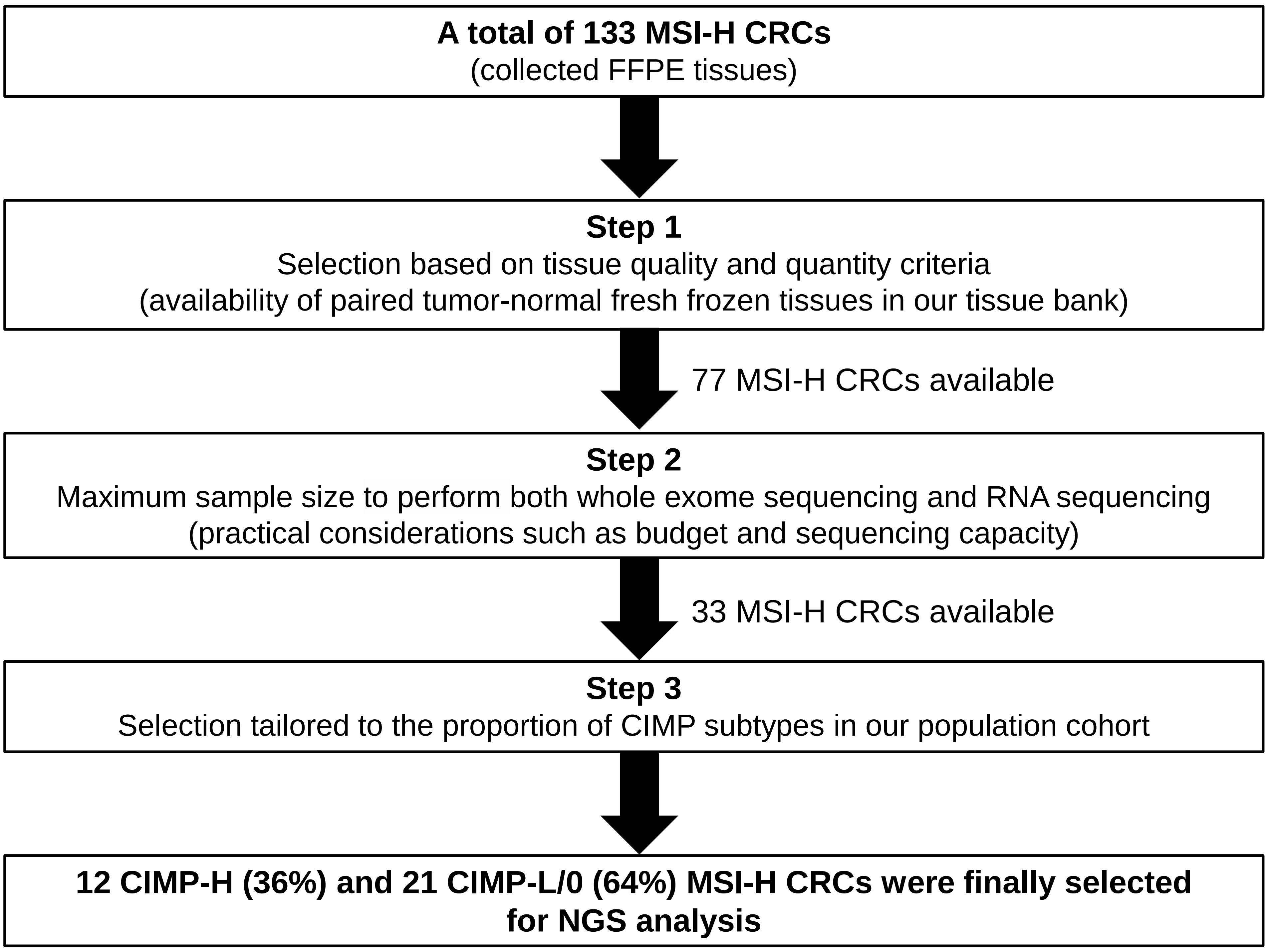

Supplementary Fig. S1 A schematic summary of sample selection steps for NGS analysis

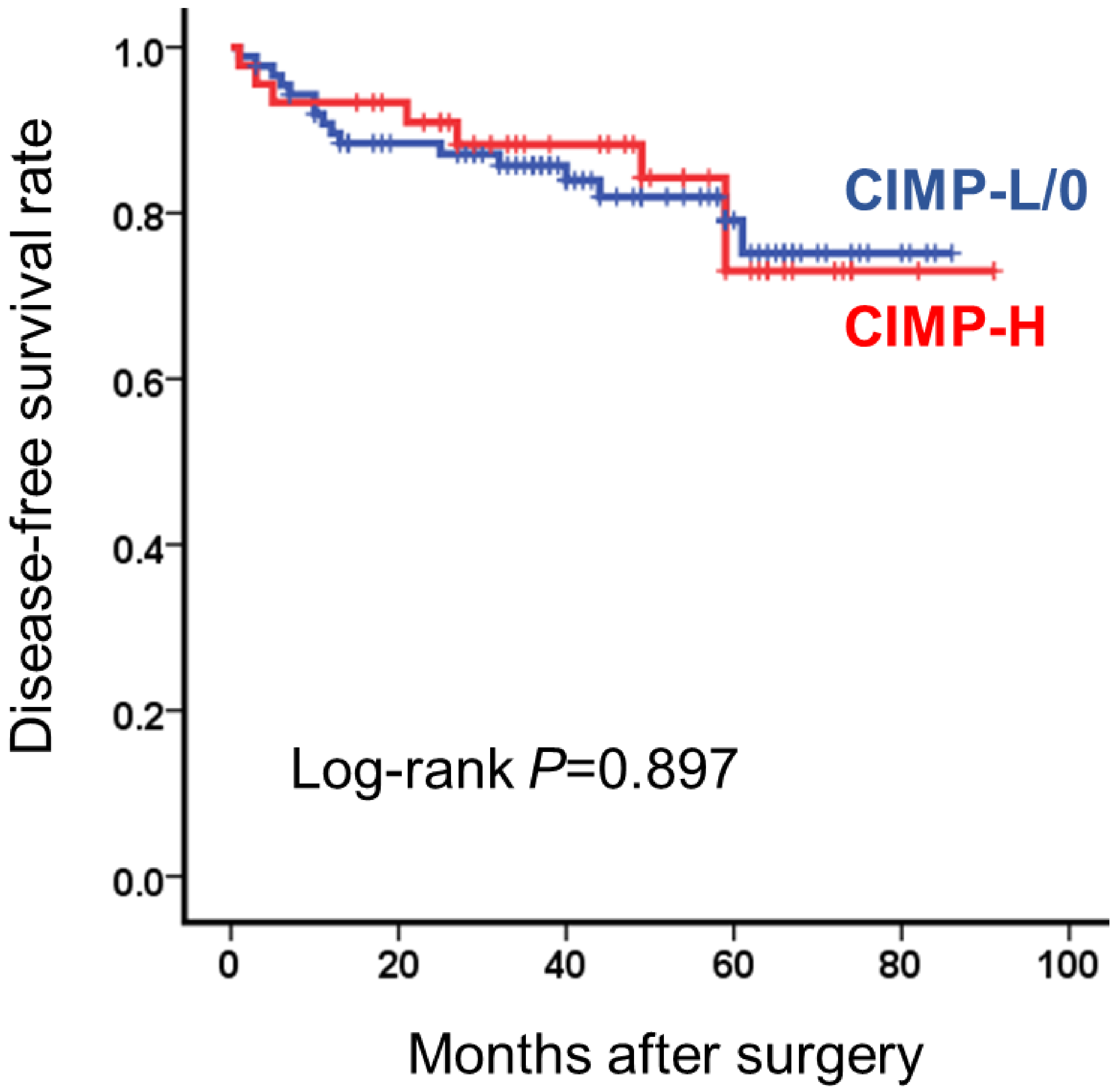

Supplementary Fig. S2 Kaplan-Meier survival analysis of patients with MSI-H CRC according to CIMP subgroups.

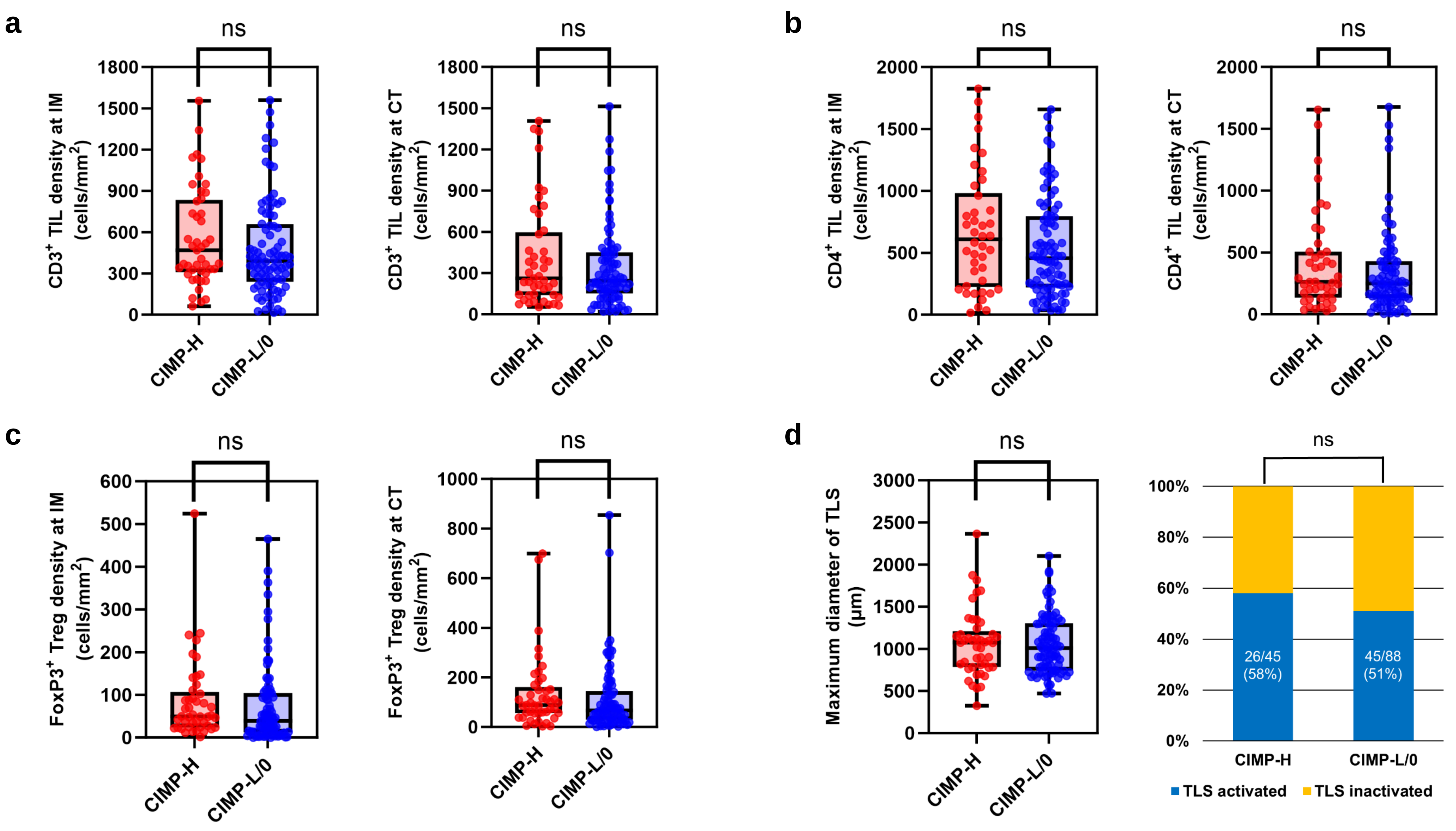

Supplementary Fig. S3 Comparison of pathology-based TIME features, including CD3+/CD4+ TILs, Treg, and TLS, between CIMP subgroups of MSI-H CRCs.

(a) Comparison of CD3+ TIL densities between CIMP-H and CIMP-L/0 subgroups of MSI-H CRCs at IM (left) and CT (right) areas.

(b) Comparison of CD4+ TIL densities between CIMP-H and CIMP-L/0 subgroups of MSI-H CRCs at IM (left) and CT (right) areas.

(c) Comparison of FoxP3+ Treg densities between CIMP-H and CIMP-L/0 subgroups of MSI-H CRCs at IM (left) and CT (right) areas.

(d) Comparison of TLS statuses, including maximum diameters of TLSs (left) and frequencies of TLS activation subgroups (right), between CIMP-H and CIMP-L/0 subgroups of MSI-H CRCs.

Abbreviations: TIME, tumor immune microenvironment; CIMP, CpG island methylator phenotype; CIMP-H, CIMP-high; CIMP-L/0, CIMP-low/negative; MSI-H, microsatellite instability-high; CRCs, colorectal cancers; TIL, tumor-infiltrating lymphocyte; Treg, regulatory T cell; IM, invasive margin; CT, center of tumor; TLS, tertiary lymphoid structure.

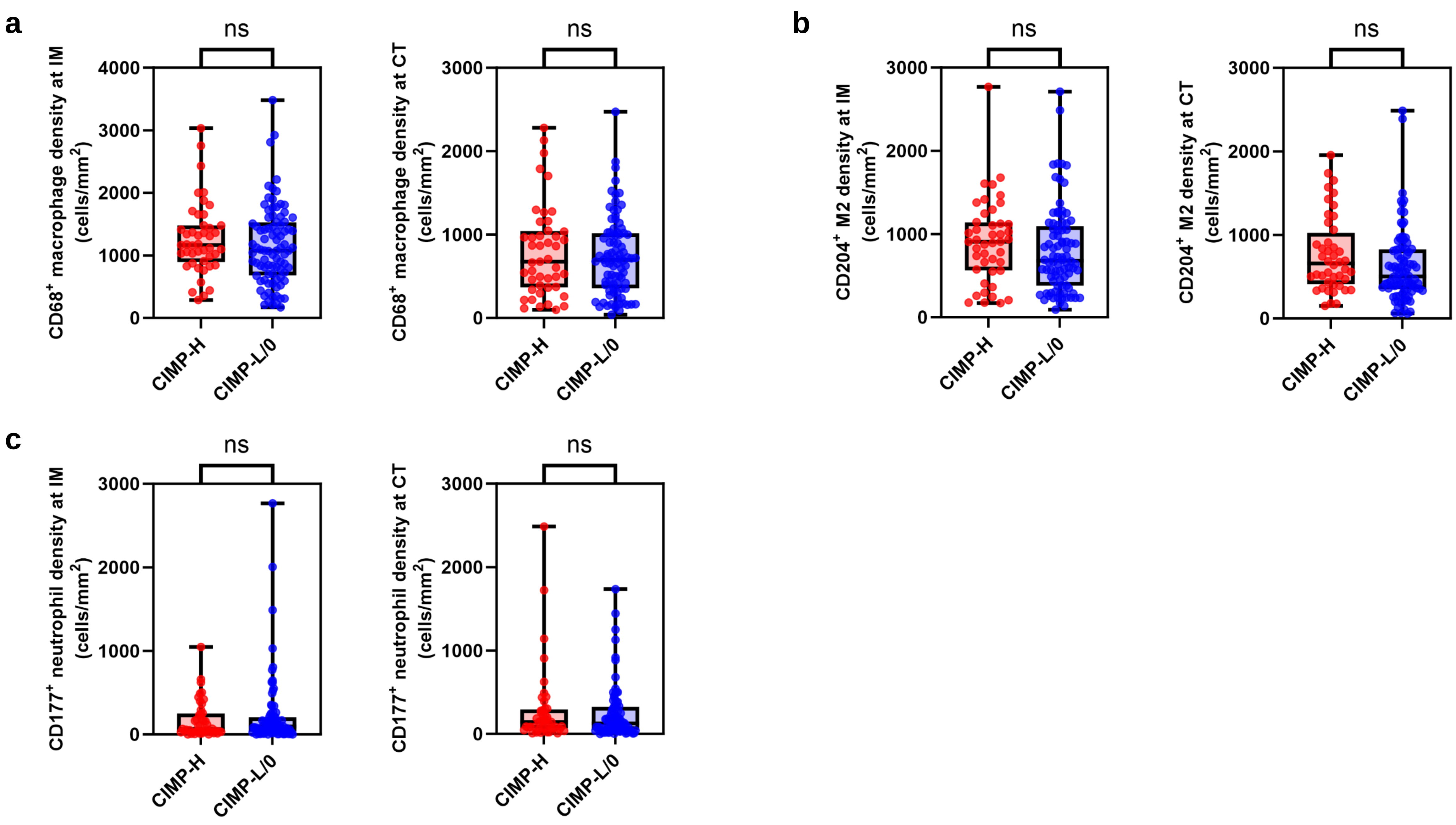

Supplementary Fig. S4 Comparison of pathology-based TIME features, including tumor-associated macrophages and neutrophils, between CIMP subgroups of MSI-H CRCs.

(a) Comparison of $CD68^+$ TAM densities between CIMP-H and CIMP-L/0 subgroups of MSI-H CRCs at IM (left) and CT (right) areas.

(b) Comparison of $CD204^+$ M2 TAM densities between CIMP-H and CIMP-L/0 subgroups of MSI-H CRCs at IM (left) and CT (right) areas.

(c) Comparison of $CD177^+$ TAN densities between CIMP-H and CIMP-L/0 subgroups of MSI-H CRCs at IM (left) and CT (right) areas.

Abbreviations: TIME, tumor immune microenvironment; CIMP, CpG island methylator phenotype; CIMP-H, CIMP-high; CIMP-L/0, CIMP-low/negative; MSI-H, microsatellite instability-high; CRCs, colorectal cancers; TAM, tumor-associated macrophage; TAN, tumor-associated neutrophil; IM, invasive margin; CT, center of tumor.

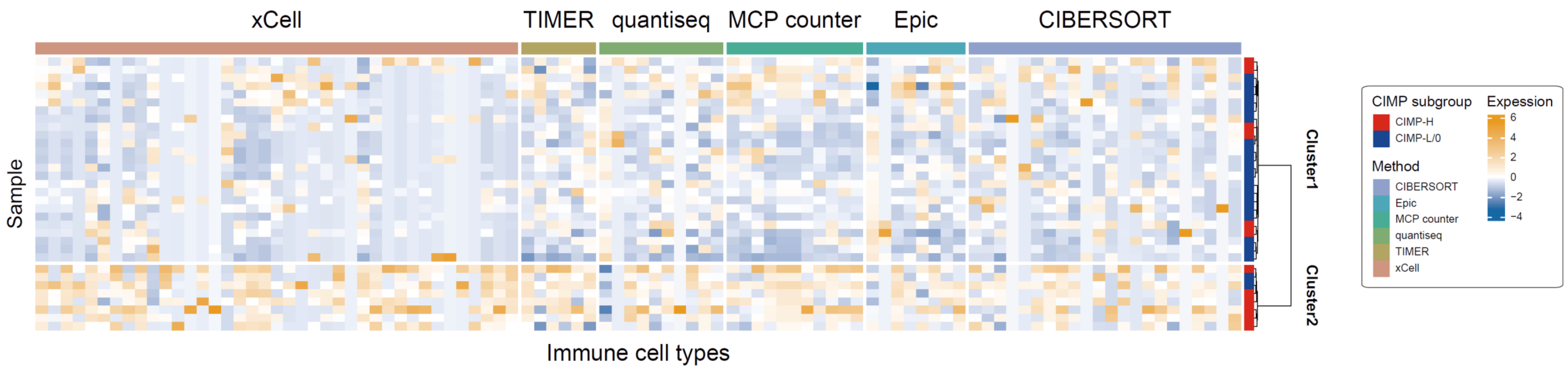

Supplementary Fig. S5 Unsupervised clustering of TIME features of MSI-H CRCs based on six algorithms for transcriptome level-based immune cell deconvolution.

Abbreviations: TIME, tumor immune microenvironment; MSI-H, microsatellite instability-high; CRCs, colorectal cancers.

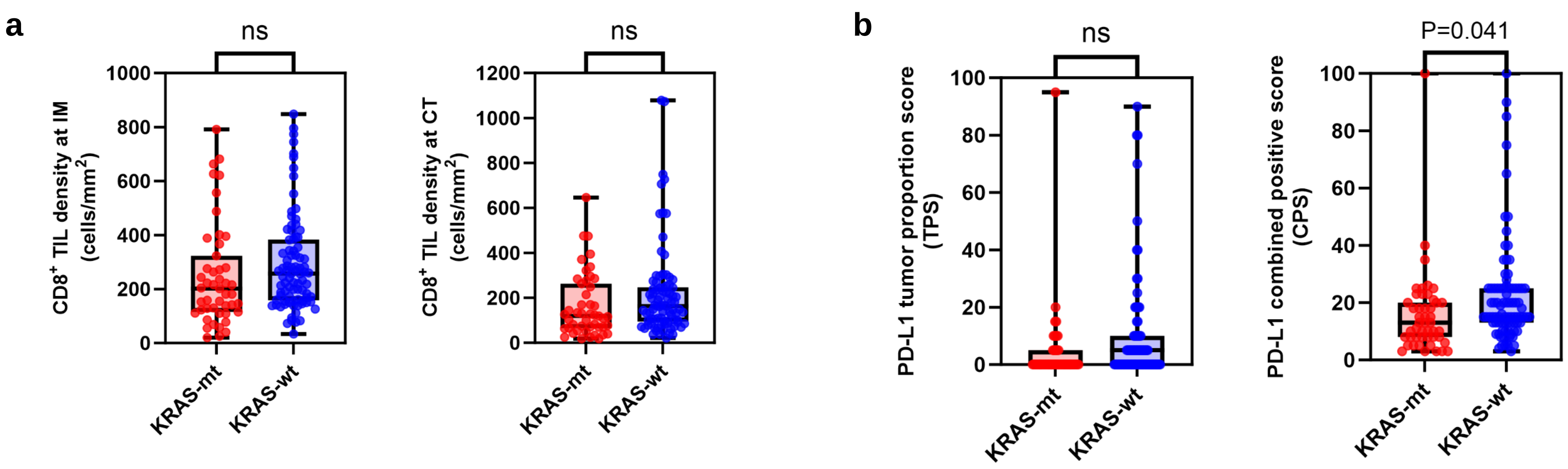

Supplementary Fig. S6 Comparison of major immune microenvironmental features according to *KRAS* mutation status in MSI-H CRCs.

(a) Comparison of $CD8^+$ TIL densities between *KRAS*-mutated and *KRAS* wild-type subgroups of MSI-H CRCs at IM (left) and CT (right) areas.

(b) Comparison of PD-L1 IHC scores, including TPS (left) and CPS (right), between *KRAS*-mutated and *KRAS* wild-type subgroups of MSI-H CRCs.
