# Supplementary material for: Immune microenvironmental heterogeneity according to tumor DNA methylation phenotypes in microsatellite instability-high colorectal cancers
Source: Cancer Immunol Immunother. 2024 Sep 5;73(11):215. doi: 10.1007/s00262-024-03805-3 (PMC11377388; doi:10.1007/s00262-024-03805-3)
Supplement: Supplementary file 2 — Supplementary file2 (PDF 177 KB) [file 262_2024_3805_MOESM2_ESM.pdf]

Supplementary Table S1. Clinicopathologic and molecular characteristics of MSI-H CRCs according to CIMP status (n = 133)

Variable		CIMP-H (n = 45)	CIMP-L/0 (n = 88)	*p*-value
Age	Older (≥ 64 years)	39 (87%)	39 (44%)	<0.001
	Younger (< 64 years)	6 (13%)	49 (56%)	
Sex	Male	11 (24%)	54 (61%)	<0.001
	Female	34 (76%)	34 (39%)	
Tumor location	Right-sided colon	41 (91%)	62 (70%)	0.007
	Left-sided colorectum	4 (9%)	26 (30%)	
Gross tumor type	Polypoid or fungating	28 (62%)	49 (56%)	0.47
	Ulceroinfiltrative	17 (38%)	39 (44%)	
Tumor size	Larger (≥ 6.4 cm)	20 (44%)	38 (43%)	0.89
	Smaller (< 6.4 cm)	25 (56%)	50 (57%)	
AJCC/UICC cancer stage	Stage I/II	33 (73%)	61 (69%)	0.63
	Stage III/IV	12 (27%)	27 (31%)	
Depth of invasion (pT)	Submucosa or proper muscle (pT1/pT2)	6 (13%)	12 (14%)	0.961
	Beyond the proper muscle (pT3/pT4)	39 (87%)	76 (86%)	
Lymph node metastasis (pN)	Absent (pN0)	34 (76%)	63 (72%)	0.626
	Present (pN1/pN2)	11 (24%)	25 (28%)	
Distant metastasis (pM or cM)	Absent (M0)	44 (98%)	79 (90%)	0.163
	Present (M1)	1 (2%)	9 (10%)	
Lymphatic invasion	Absent	26 (58%)	67 (76%)	0.029
	Present	19 (42%)	21 (24%)	
Venous invasion	Absent	42 (93%)	74 (84%)	0.131
	Present	3 (7%)	14 (16%)	
Perineural invasion	Absent	36 (80%)	63 (72%)	0.293
	Present	9 (20%)	25 (28%)	
Tumor grade (histologic differentiation)	Low-grade (well or moderately differentiated)	27 (60%)	60 (68%)	0.348
	High-grade (poorly differentiated)	18 (40%)	28 (32%)	

Mucinous histology	Non-mucinous (< 50%)	34 (76%)	63 (72%)	0.626
	Mucinous (≥ 50%)	11 (24%)	25 (28%)	
Medullary histology	Non-medullary (< 50%)	32 (71%)	80 (91%)	0.003
	Medullary (≥ 50%)	13 (29%)	8 (9%)	
Signet ring cell histology	Absent	40 (89%)	74 (84%)	0.454
	Present (≥ 5%)	5 (11%)	14 (16%)	
Tumor budding	BD1 or BD2 (low or intermediate)	31 (69%)	66 (75%)	0.453
	BD3 (high)	14 (31%)	22 (25%)	
Poorly differentiated clusters	G1 or G2 (low or intermediate)	25 (56%)	51 (58%)	0.791
	G3 (high)	20 (44%)	37 (42%)	
Desmoplastic reaction	Mature or intermediate	42 (93%)	84 (95%)	0.688
	Immature	3 (7%)	4 (5%)	
MLH1 expression	Loss	45 (100%)	41 (47%)	<0.001
	Retained	0 (0%)	46 (53%)	
MSH2 expression	Loss	0 (0%)	18 (21%)	0.001
	Retained	45 (100%)	69 (79%)	
MSH6 expression	Isolated loss	0 (0%)	17 (19%)	0.002
	Retained or non-isolated loss	45 (100%)	71 (81%)	
PMS2 expression	Isolated loss	0 (0%)	12 (14%)	0.008
	Retained or non-isolated loss	45 (100%)	76 (86%)	
KRAS mutation	Absent	42 (93%)	44 (50%)	<0.001
	Present	3 (7%)	44 (50%)	
BRAF mutation[a]	Absent	32 (73%)	87 (100%)	<0.001
	Present	12 (27%)	0 (0%)	

Abbreviations: MSI-H, microsatellite instability-high; CRCs, colorectal cancers; CIMP, CpG island methylator phenotype; CIMP-H, CIMP-high; CIMP-L/0, CIMP-low/negative; AJCC/UICC, American Joint Committee on Cancer/Union for International Cancer Control

[a]Two cases were excluded from *BRAF* mutation analysis due to suboptimal quality or quantity of their isolated DNA samples.
